# Trophy Hunting and Sustainability: Temporal Dynamics in Trophy Quality and Harvesting Patterns of Wild Herbivores in a Tropical Semi-Arid Savanna Ecosystem

**DOI:** 10.1371/journal.pone.0164429

**Published:** 2016-10-13

**Authors:** Victor K. Muposhi, Edson Gandiwa, Paul Bartels, Stanley M. Makuza, Tinaapi H. Madiri

**Affiliations:** 1 School of Wildlife, Ecology and Conservation, Chinhoyi University of Technology, Private Bag 7724, Chinhoyi, Zimbabwe; 2 Department of Nature Conservation, Tshwane University of Technology, Private Bag X680, Pretoria, 0001, South Africa; 3 School of Agricultural Sciences and Technology, Chinhoyi University of Technology, Private Bag 7724, Chinhoyi, Zimbabwe; 4 Zimbabwe Parks and Wildlife Management Authority, PO Box CY140, Causeway, Harare, Zimbabwe; Fred Hutchinson Cancer Research Center, UNITED STATES

## Abstract

The selective nature of trophy hunting may cause changes in desirable phenotypic traits in harvested species. A decline in trophy size of preferred species may reduce hunting destination competitiveness thus compromising the sustainability of trophy hunting as a conservation tool. We explored the trophy quality and trends in harvesting patterns (i.e., 2004–2015) of Cape buffalo (*Syncerus caffer)*, African elephant (*Loxodonta africana*), greater kudu (*Tragelaphus strepsiceros)* and sable (*Hippotragus niger*) in Matetsi Safari Area, northwest Zimbabwe. We used long-term data on horn and tusk size, age, quota size allocation and offtake levels of selected species. To analyse the effect of year, area and age on the trophy size, quota size and offtake levels, we used linear mixed models. One sample *t*-test was used to compare observed trophy size with Safari Club International (SCI) minimum score. Trophy sizes for Cape buffalo and African elephant were below the SCI minimum score. Greater kudu trophy sizes were within the minimum score threshold whereas sable trophy sizes were above the SCI minimum score between 2004 and 2015. Age at harvest for Cape buffalo, kudu and sable increased whilst that of elephant remained constant between 2004 and 2015. Quota size allocated for buffalo and the corresponding offtake levels declined over time. Offtake levels of African elephant and Greater kudu declined whilst the quota size did not change between 2004 and 2015. The quota size for sable increased whilst the offtake levels fluctuated without changing for the period 2004–2015. The trophy size and harvesting patterns in these species pose a conservation and management dilemma on the sustainability of trophy hunting in this area. We recommend: (1) temporal and spatial rotational resting of hunting areas to create refuge to improve trophy quality and maintenance of genetic diversity, and (2) introduction of variable trophy fee pricing system based on trophy size.

## Introduction

Wildlife conservation is characterised by proprietorship and pricing systems within the biological parameters that limit sustainable utilization [1, 2]. This is opposed to wildlife preservation approaches which promote restraint in the harvest and consumption of wildlife species and their products [3]. In sub-Saharan Africa, wildlife conservation in protected areas [4] is substantially supported by revenue generated through sustainable harvesting of wildlife species through trophy hunting in Category VI protected areas [5, 6]. Trophy hunting refers to hunting by paying tourists, typically with the objective of selecting individuals with exceptional phenotypic traits (e.g., large horns, tusks, body size, mane or skull length) and usually in the company of a professional hunting guide [7]. Though there has been perpetual debate and polarity on the sustainability of trophy hunting in most sub-Saharan African countries [8], it is still considered as one sustainable way of supporting conservation in African countries endowed with abundant wildlife species [9].

In this study, sustainability refers to the ability of trophy hunting to support, sustain and ensure persistence of wildlife populations without compromising their abundance and diversity over a long-term within the framework of intergenerational equity [10, 11]. In inaccessible, remote and marginalised areas lacking infrastructure, attractive scenery or high densities of charismatic and viewable wildlife species, trophy hunting is considered more suitable and feasible alternative for revenue generation over other forms of tourism (i.e., ecotourism, photographic tourism, enclave tourism [12]) [13, 14]. It is also becoming evident that trophy hunting may provide revenue generation for conservation opportunities in countries where other forms of tourism may not be suitable due political instability [15] and negative media framing [16].

Central to the controversy of trophy hunting is the continual decline and possible expiation of wildlife populations in most sub-Saharan countries [17–19]. Historically, unregulated hunting in some continents led to the extinction of some wildlife species through what has been referred to as the global blitzkrieg (overkill) hypothesis [20, 21], whilst the African continent was to some extent spared from this unprecedented loss of species due to over harvesting and illegal hunting activities [22]. In recent times, declines in wildlife populations globally (including Africa) have been associated with among others, illegal hunting [23–28], over harvesting [29–31], droughts [32–34] and fragmented and weak hunting policies that regulate harvesting of wildlife species [35]. However, trophy hunting uses a quota system approach that promotes sustainable off-takes by harvesting small portions of the natural population growth rates which arguably falls within the compensatory mortality range and has a negligible impact on overall ecology of wildlife species [36, 37].

A quota refers to the number of individuals of a particular animal species that is legally allocated or prescribed for harvesting per year for a particular area [38]. The quota system used is based on ecological theory, i.e., maximum sustainable yield (MSY), set in such a way that off-take levels are always below the growth rate of the target species at any given time [39, 40]. Accordingly, trophy hunting is meant to remove only a few individuals, mostly those that have passed their prime reproductive time and as such should not compromise viability of wildlife species [41–43].

The size of a quota allocated for trophy hunting is mostly influenced by several factors such as the population size [39], trophy size, hunting success [44], age at harvest [45], habitat management and whether or not the populations are shared by two or more management regimes (e.g. in the context of KAZA TFCA). The frequency at which these factors are monitored and analysed to inform the quota setting process as part of the adaptive management process is often low and not consistent [41, 46]. Fragmented monitoring programs of these parameters are mostly a result of the long-term costs associated with their monitoring over time, thus compromising the effectiveness of the process [47]. Moreover, the use of MSY tends to be problematic as it has been developed and mostly utilised in aquatic ecosystems where severe declines in important fish catches have been witnessed [48, 49]. Though the MSY concept is theoretically sound, its applicability in reality is marred with several challenges such as politics, fixed quotas, shared populations which may ultimately cause declines in wildlife species [50, 51].

The sustainability of trophy hunting in Category VI protected areas in most southern African countries is increasingly subjected to scrutiny both from an ecological and ethical perspective [52]. It is becoming evident that there are some negative effects of trophy hunting on the phenotypic traits and population dynamic of hunted species. Some studies have shown that selective harvesting related to trophy hunting may result in the loss of the more desirable phenotypic traits (i.e., horn or tusk size) with increasing hunting pressure [53]. Despite these observations, there are few studies reporting on the decline of trophy size in hunting destinations in southern Africa, e.g., Zambia [54], northwest Zimbabwe [55], and South Africa [56, 57]. However, the declines in horn size cannot be attributed solely to selective hunting pressure let alone inbreeding depression but a combination of these with some environmental factors [58]. Nonetheless, little attention has been given to establish the relationship between observed trophy size and the standard trophy size of harvested species [44].

In Zimbabwe, trophy hunting mainly occurs in safari areas, communal areas and private areas [59]. The Zimbabwe Parks and Wildlife Management Authority administers a participatory quota setting system with the concerned stakeholders (i.e., private land owners, communal areas representatives and private concessionaires in state owned safari areas) as a way of controlling the offtake levels through trophy hunting. The north-western side of Zimbabwe is covered by Matetsi Safari Area, one of the prime hunting areas known to have conservative and considerably low quota allocations for some wildlife species [51]. These low quotas (< 5% of the target population size) are believed to have a considerable effect on the population size let alone the horn or tusk size of targeted species [60].

Conservationists argue that there is much uncertainty over the sustainability of offtake rates and their potential impacts on wildlife populations. For instance, the United States of America has taken bold steps in banning import of ivory and related products especially from Kenya and Zimbabwe since 2014. Coincidentally, some commercial passenger and cargo airlines have also put in place an embargo on the transportation of trophies of legally and sustainably hunted species [9]. These embargoes have been worsened by the negative and emotive media framing of trophy hunting in Zimbabwe following the controversial killing of ‘Cecil’ the lion (*Panthera leo*) by an American hunting tourist near Hwange National Park [8]. This negative media framing of a country may reduce its attractiveness as a destination, which result in low offtake levels of species thus reducing revenue generation from trophy hunting [16]. Furthermore, considering the negative media framing of Zimbabwe during the period of political instability and economic decline, 2000–2008 [16], as well as restrictive policy on trophy imports [9], international trophy hunters may avoid Zimbabwe as a hunting destination thus reducing the trophy hunting offtake levels in hunting areas compared to the period of political inclusiveness and economic recovery (2009–2015).

Although several studies have been done on trophy hunting of lions and leopards (*Panthera pandus*) [46, 61–64], few studies have explored on trophy size related issues on large wild herbivores in southern Africa [44, 55–57]. In this study, we explored the temporal dynamics in trophy quality and harvesting patterns of four selected wild herbivores, Cape buffalo (*Syncerus caffer)*, African elephant (*Loxodonta africana*) and mid-sized herbivores, greater kudu (*Tragelaphus strepsiceros)* and sable (*Hippotragus niger*) in a semi arid tropical ecosystem, Matetsi Safari Area, a hunting complex within the Kavango Zambezi Transfrontier Conservation Area (KAZA TFCA), northwest of Zimbabwe. We tested three hypotheses, (1) selective harvesting through trophy hunting may result in reduced horn or tusk size and age at harvest of selected wild herbivores with the passage of time, (2) sustainable utilization management programs may reduce the quota size allocated for selected wild herbivores and their offtake levels over time commensurate with the population and trophy size trends in different hunting areas, and (3) economic status of the country between the period 2004–2015 would have an effect on quota size and offtake levels in Matetsi Safari Area, Zimbabwe.

## Methods

### Study Area

**Fig 1:**
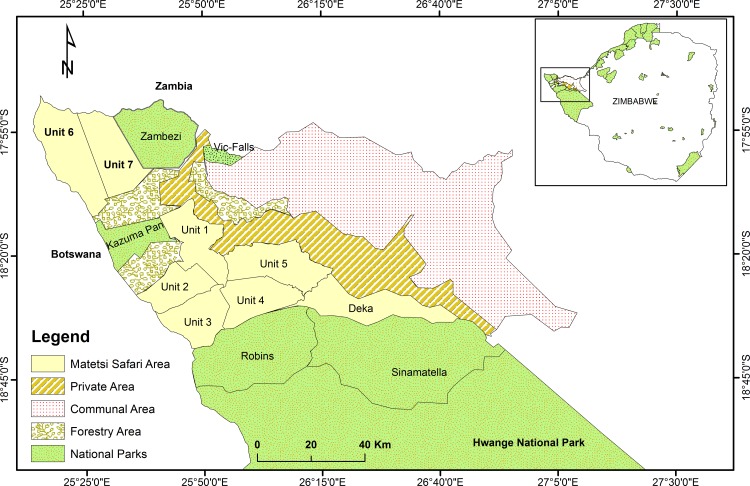
Map showing location of study area, Matetsi Safari Area and the surrounding areas (National Parks, Forestry Areas, Private Areas and Communal Areas in northwest Zimbabwe). Insert: Location of study area (solid rectangle) in Zimbabwe in relation to other protected areas Source: Muposhi, Gandiwa [70].

The study was conducted in an unfenced protected area network, Matetsi Safari Area that covers approximately 3,000 km^2^, northwest of Zimbabwe (Fig 1). Matetsi Safari Area is part of the Kavango Zambezi Transfrontier Conservation Area (KAZA TFCA) which is shared between Angola, Botswana, Namibia, Zambia and Zimbabwe established in 2011 [65, 66]. In Zimbabwe, Protected Areas Category VI is referred to as Safari Areas. These areas occur mainly surrounding National Parks and are managed mainly for the sustainable use of natural ecosystems and as part of a buffer zone to cushion National Parks from human disturbances [4]. Matetsi Safari Area is divided into seven hunting management blocks called Units (Table 1).

**Table 1:** Characteristics of the seven management Units, (i.e., hunting status and estimated area), of Matetsi Safari Area, Zimbabwe.

Unit	Concession holder	Hunting status	Area (km^2^)
1	*Private concession	Since 1973	398
2	††ZimParks	Since 2013	475
3	Private concession	Since 1973	293
4	ZimParks	Since 2012	358
5	ZimParks	Since 2005	364
6	Private concession	Since 1973	592
7	Non-hunting concession	^#^Since 1973	447

Notes:

††ZimParks stands for Zimbabwe Parks and Wildlife Management Authority.

*Private concession here refers to a medium to long-term lease given to a private outfitter by the Zimbabwe Parks and Wildlife Management Authority to conduct hunts in a Safari Area within the Parks and Wildlife Estate.

^#^A non-hunting private concession, mostly photographic tourism and ecotourism.

The southern block (i.e., comprise of Unit 1–5) is boarded by Hwange National Park to the southern part, north-eastern side with private and communal areas whereas the western side is mostly Kazuma Pan National Park and Forestry Area Hunting block. However, the northern block (i.e. Unit 6 and 7), are sandwiched by protected areas, Zambezi National Park to the eastern side, and to the western side are Chobe National Park, Botswana and Forestry Area to the south. Trophy hunting has been the sole land use option for Matetsi Safari Area for more than 37 years [67]. The main soil types on sites are lithosols and regosols occurring on Karoo volcanic and Kalahari geological formations, respectively [68]. The lithosols are dominated by *Colophospermum mopane* and *Terminalia species*. [69] whilst *Baikiaea plurijuga*, which occurs in association with *Pterocarpus angolensis* and *Guibortia coleosperma* dominate on the regosols [68]. Some of the common wildlife species in the study area include: herbivores (African buffalo, Burchell’s zebra (*Equus quagga*), elephant, giraffe (*Giraffa camelopardalis*), greater kudu, impala (*Aepyceros melampus*), reedbuck (*Redunca arundinum*), sable antelope, warthog (*Phacochoerus aethiopicus*) and waterbuck (*Kobus ellipsiprymnus*), wildebeest (*Connochaetes taurinus*)) and carnivores (leopard, Lion, Hyena (*Crocuta crocuta*)).

#### Study species

The following wild herbivores were used as study species for this study: mega herbivores, Cape buffalo, elephant and medium-sized herbivores, greater kudu *and* sable. These four species were selected on the basis that they are amongst the most commonly hunted herbivores in southern Africa [13, 44, 71]. In addition, complete records on trophy size, quota allocations and utilization levels for the species were readily available for the period 2004–2015 at Matetsi Safari Area headquarters. The densities of these species in this area have been documented by Crosmary, Côté [51] as: Cape buffalo (1.4 individuals per km^2^), African elephant (0.7 individuals per km^2^), greater kudu (1.4 individuals per km^2^) and sable (0.7 individuals per km^2^) for the period 1995–2010. During this same period, the average harvest rates (i.e., number of individuals harvested per year divided by the total population estimate for that year [51]) was 1.7 ± 1.2% for the four species.

### Data Collection

We collected long-term data for the six hunting units (i.e. Unit 1–6, see Table 1), from Matetsi Safari Area headquarters on; (1) trophy size and age at harvest data for the period 2004–2015, and (2) annual quotas allocation and offtake levels for African elephant, Cape buffalo, greater kudu and sable for the period 2004–2015 for Unit 1–6 (S1 and S2 Files). All trophy size measurements for the four species were done by parks rangers following the Safari Club International (SCI) scoring system (http://www.scirecordbook.org/docs/methods). The associated data on age at harvest for the four wild herbivores was estimated by parks rangers using dentition and jaws [72–74] as part of their monitoring routine at Matetsi Safari Area. We considered the number of animals harvested off an allocated quota for each year as the offtake level. We further determined offtake growth rate per species, i.e., mean annual change in offtake size, which was calculated using the following formulae after Rist, Milner-Gulland [75]:

$$Log\;\left(h_{t+1}\right)–Log\;\left(h_{t}\right);\;where\;h_{t}is\;the\;size\;of\;the\;total\;offtake\;in\;year\;t.$$

We expected that the offtake rates would be analogous to population size and happens to be positively correlated [76], thus can be used as proxy or index for population estimates of harvested species. Accordingly, we used data of annual utilization of the quota allocation for each hunting unit for the period 2004–2015 except for the African elephant with an incomplete data set so we used data for 2005–2015.

#### Data analysis

Data on trophy size, age at harvest, quota size and offtake levels were tested for normality and equality of variance to using Shapiro Wilk test and Levene’s test respectively to ascertain if the normality assumptions were being satisfied. All data on explanatory variables, i.e., trophy size, age at harvest, quota size and offtake level were found to conform to the normality assumptions. We grouped data on quota size and offtake levels into two time intervals based on the temporal economic status: (a) period of land reform, hyper inflation and policy changes, 2004–2009, and (b) period of political stability, deflation and economic recovery, 2009–2015, in Zimbabwe. All the data were analysed separately for each species.

First, we computed a simple linear regression to assess the temporal trends in the trophy size and age at harvest for the four herbivores. A linear mixed model (LMM) was used to analyze the variation in trophy size, age at harvest and offtake levels trends for the six hunting units for the period 2004–2015. The model parameters included the trophy size as the dependant variable whilst year and area were fixed variables and the age at harvest being the covariate. We further conducted a one-sample *t*-test to ascertain if the observed trophy size differed from the SCI minimum score for greater kudu (121 inches), sable (96 inches), Cape buffalo (101 inches) and African elephant (90 pounds) (http://www.scirecordbook.org). Second, we used Analysis of Variance (ANOVA) to assess the spatial variation in quota size and trophy size of the selected herbivores across the six hunting units. Significant effects were followed by pair-wise contrasts using sequential Bonferroni *post hoc* adjusted significance (p < 0.05). Third, to establish the effect of temporal economic status on harvesting patterns, we computed an independent *t*-test to compare the overall quota size and offtake levels for the two time periods, i.e., 2004–2008 and 2009–2015. We conducted all statistical analyses in IBM SPSS 20 software package (IMB, New York, USA) at 5% level of significance.

## Results

### Trophy Size and Age at Harvest Patterns

The total number of harvested individuals for the four selected wild herbivores was: Cape buffalo: 807, greater kudu: 565 and sable: 369, for the period 2004–2015 and African elephant: 258, for 2005–2015. During the period 2004–2015, the observed mean Cape buffalo trophy size (95.39 ± 8.66 inches) were below 101 inches, the SCI minimum score (t_(806)_ = -18.41, p < 0.001). Similarly, the mean African elephant trophy size (81.40 ± 21.35 pounds) was below the SCI minimum score of 90 pounds for the period 2005–2015 (t_(257)_ = -6.47, p < 0.001). On the contrary, the mean trophy size for greater kudu (120.47 ± 7.54 inches) for the period 2004–2015 was similar to the SCI minimum score levels (t_(564)_ = -1.68, p = 0.094) of 121 inches. Of the four herbivores, only sable had a mean trophy size (98.89 ± 6.34 inches) higher than the SCI minimum score (96 inches) during the period 2004–2015 (t_(369)_ = 8.76, p < 0.001).

**Fig 2:**
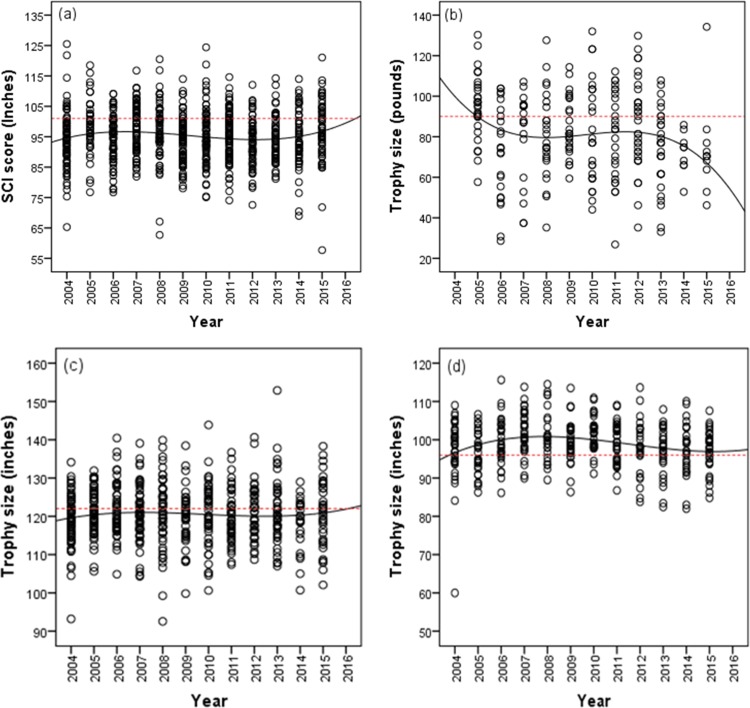
**Temporal trend in mean trophy size for the harvested wild herbivores, (a) Cape buffalo, (b) African elephant, (c) greater kudu, and (d) sable for the period 2004–2015 in Matetsi Safari Area, Zimbabwe.** *Notes*: *Solid circles indicate mean trophy size*, *solid line represents trend in trophy size*, *hollow circle indicate mean age at harvest*, *dotted broken line represent trend in age at harvest*.

**Fig 3:**
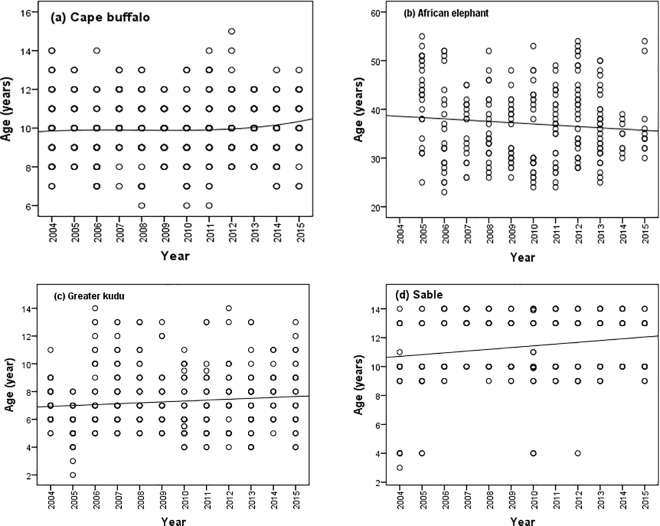
**Temporal trend in mean age at harvest of the four selected wild herbivores, (a) Cape buffalo, (b) African elephant, (c) greater kudu, and (d) sable for the period 2004–2015 in Matetsi Safari Area, Zimbabwe**.

We found no significant trends in the trophy size of Cape buffalo, Greater kudu and sable (p > 0.05) for the period 2004–2015 (Fig 2A, 2C and 2D). However, the trophy size of African elephant declined significantly (β ± SE: -1.03 ± 0.45, t = -2.29, p = 0.023) for the period 2004–2015 (Fig 2B). The effect of area was not significant though its interaction with year and age at harvest was significant for the four species during the same period (Table 2). The temporal patterns on age at harvest for Cape buffalo, greater kudu and sable recorded for the period 2004–2015 were significant (Table 3). On the contrary, African elephant age at harvest did not change over time for the period 2005–2015 (R^2^ = 0.01, β ± SE: -0.26 ± 0.17, t = -1.58, p = 0.115; Table 3, Fig 3).

**Table 2:** Linear mixed model results showing the fixed effects of year, hunting area and age at harvest on the trophy size for the period 2004–2015 in Matetsi Safari Area, Zimbabwe.

Variable	df	F-statistic	p-value
**Cape buffalo**			
Year	11	5.33	0.000
Area	5	1.09	0.363
Age	1	111.08	0.000
Year * Area	53	2.02	0.000
Year * Area * Age	69	3.64	0.000
**African elephant**			
^**††**^Year	10	1.19	0.305
Area	5	1.21	0.306
Age	1	103.73	0.000
Year * Area	42	1.43	0.064
Year * Area * Age	57	1.98	0.001
**Greater kudu**			
Year	11	8.46	0.000
Area	5	2.00	0.078
Age	1	157.17	0.000
Year * Area	50	1.57	0.010
Year * Area * Age	66	2.60	0.000
**Sable**			
Year	11	5.84	0.000
Area	5	1.41	0.222
Age	1	97.67	0.000
Year * Area	51	1.67	0.006
Year * Area * Age	67	2.78	0.000

^**††**^Data for the year 2004 was missing; only data for the period 2005–2015 was presented

**Table 3:** Model parameters (β ± SE) of temporal trends in the age at harvest for Cape buffalo, African elephant, greater kudu and sable for the period 2004–2015 in Matetsi Safari Area, Zimbabwe.

Species	β ± SE	t-value	p-value
Cape buffalo	0.03 ± 0.02	2.22	0.028
African elephant	-0.26 ± 0.17	-1.58	0.115
Greater kudu	0.16 ± 0.03	2.58	0.000
Sable	0.11 ± 0.04	3.51	< 0.001

Notes: Beta coefficient (β) shows the slope of the trend line.

**Fig 4:**
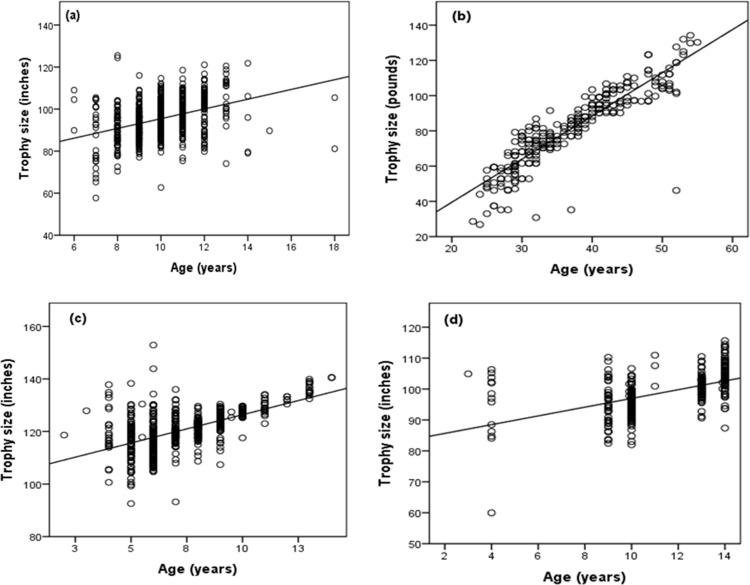
**Relationship between the trophy size and age at harvest of the four selected wild herbivores, (a) Cape buffalo, (b) African elephant, (c) greater kudu, and (d) sable for the period 2004–2015 in Matetsi Safari Area, Zimbabwe**.

There was a positive relationship between the age at harvest and the trophy size for all the harvested herbivore species for the period 2004–2015, i.e. cape buffalo (R^2^ = 0.15, β(SE): 2.32(0.20), t = 11.71, p < 0.001, Fig 4A), African elephant (R^2^ = 0.80, β(SE): 2.45(0.08), t = 32.00, p < 0.001, Fig 4B), greater kudu (R^2^ = 0.33, β(SE): 2.15(0.13), t = 16.80, p < 0.001, Fig 4C) and sable (R^2^ = 0.28, β(SE): 1.41(0.12), t = 11.87, p < 0.001, Fig 4D).

For the period 2004–2015, only greater kudu mean age at harvest and trophy size did not vary with the hunting Unit, i.e., age at harvest (F_(5, 560)_ = 1.35, p = 0.385) and trophy size (F_(5, 560)_ = 0.24, p = 0.859) (Table 4). Bonferroni *post hoc* test however showed that the trophy size and age at harvest for individuals in Unit 6 were higher than the other Units except for Cape buffalo (Table 4).

**Table 4:** Estimated marginal means (±SD) of age at harvest and the trophy size for Cape buffalo, African elephant, greater kudu and sable observed for the period 2004–2015 for the six hunting units in Matetsi Safari Area, Zimbabwe.

Unit	Cape buffalo		African elephant		Greater kudu		Sable	
	Age (years)	Trophy size (inches)	Age (years)	Trophy size (pounds)	Age (years)	Trophy size (inches)	Age (years)	Trophy size (inches)
1	9.96 ± 1.35	95.94 ± 8.15	32.95 ± 6.42	68.53 ± 20.03	7.34 ± 2.11	121.27 ± 7.34	10.98 ± 2.44	98.46 ± 6.44
2	9.90 ± 1.36	93.56 ± 9.35	38.21 ± 7.27	86.35 ± 17.04	7.26 ± 1.99	119.56 ± 7.29	11.81 ± 2.39	99.90 ± 6.56
3	10.04 ± 1.53	97.39 ± 9.14	38.84 ± 7.16	85.81 ± 19.08	7.40 ± 1.89	120.13 ± 7.13	11.08 ± 2.18	99.11 ± 5.63
4	10.15 ± 1.51	95.77 ± 7.78	34.66 ± 7.53	75.30 ± 21.15	7.24 ± 2.27	120.23 ± 7.83	11.23 ± 2.63	97.08 ±7.99
5	10.25 ± 1.50	95.42 ± 8.86	36.15 ± 8.23	78.67 ± 22.16	7.06 ± 1.83	119.67 ± 8.02	11.00 ± 2.00	97.04 ± 5.50
6	9.61 ± 1.26	93.73 ± 8.26	41.37 ± 7.24	93.15 ± 19.33	7.14 ± 2.15	121.85 ± 7.70	12.33 ± 2.32	101.6 ± 4.93
F-statistic	3.18	3.758	7.24	8.22	1.35	0.242	3.27	4.35
p-value	0.008	0.002	< 0.001	< 0.001	0.385	0.859	0.007	0.001

Notes: Safari Club International minimum scores, Cape buffalo: 101 inches, African elephant: 90 pounds, greater kudu 121 inches and sable: 96 inches.

### Temporal and Spatial Harvesting Patterns

**Fig 5:**
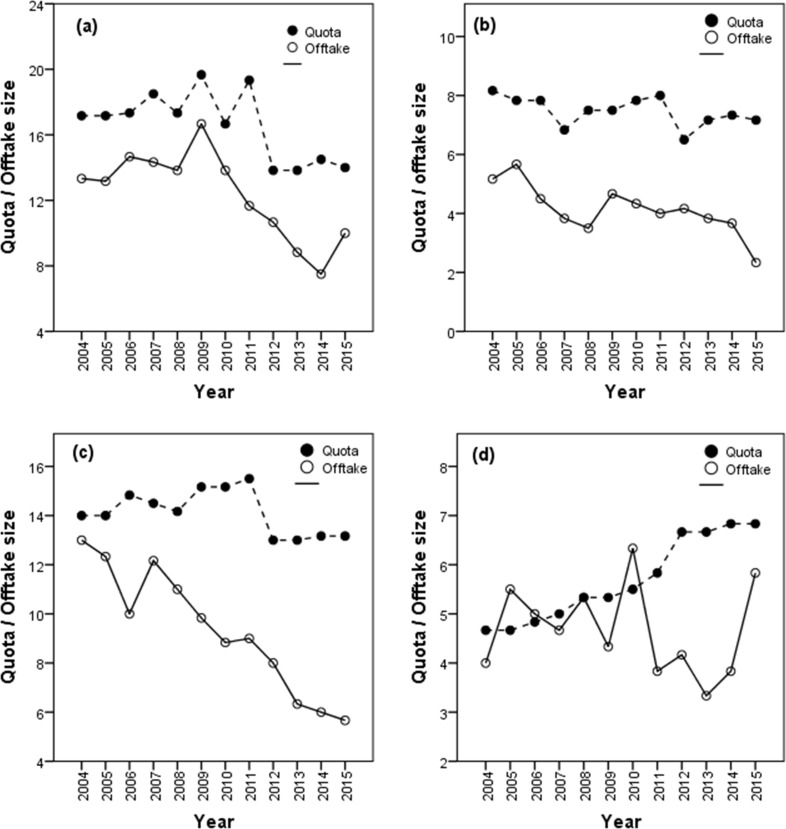
**Temporal patterns in quota size and offtake level for (a) Cape buffalo, (b) African elephant, (c) greater kudu, and (d) sable for the period 2004–2015 in Matetsi Safari Area, Zimbabwe.** Notes: solid line with solid circles show the quota size trend; dotted line with hollow circle show trend in offtake levels.

The annual quota allocated for Cape buffalo for the period 2004–2015 declined (β ± SE: -0.36 ± 0.14, t = -2.52, p = 0.014) which corresponded to a decline in the offtake levels (-0.55 ± 0.21, t –2.63, p < 0.000, Fig 5A) in Matetsi Safari Area. However, no significant changes were recorded in the allocated African elephant quota (-0.07 ± 0.05, t = -1.51, p = 0.137) whilst the offtake levels declined (-0.18 ± 0.08, t = -2.37, p = 0.021) for the period 2004–2015 (Fig 5B). Similarly, significant declines in the Greater kudu offtake (-0.67 ± 0.14, t = -4.91, p < 0.000) were recorded whereas the quota size did not change (-0.12 ± 0.09, t = -1.13, 0.224, Fig 5C) for the period 2004–2015. On the contrary, the period 2004–2015 in Matetsi Safari Area was characterized with an increase in the annual quota allocation for sable antelope (0.23 ± 0.04, t = 6.27, p < 0.000) whilst the fluctuations on the offtake levels over time were non-significant (-0.04 ± 0.10, t = -0.39, p = 0.697, Fig 5D).

### Effect of Temporal Economic Status Harvesting Regime Patterns

**Fig 6:**
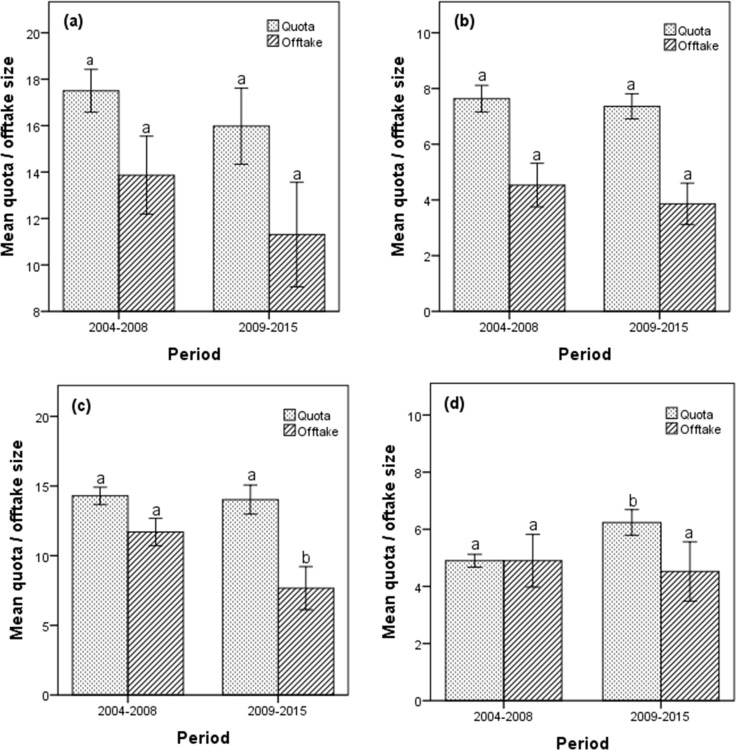
**Observed mean quota and offtake size for (a) Cape buffalo, (b) African elephant, (c) greater kudu and, (d) sable antelope for the two periods, 2004–2009 and 2009–2015 in Matetsi Safari Area, Zimbabwe.** Notes: error bars show the 95% confidence intervals; different superscript (a, b) in the same category denotes significant differences, p < 0.05.

The period 2004–2015 was characterized by significant changes in the offtake growth rate for Cape buffalo (F_(11, 60)_ = 2.01, p = 0.043). However, we recorded no difference in the quota size (t_(70)_ = 1.47, p = 0.145) of Cape buffalo between the period 2004 and 2008 and 2009–2015 as well as the offtake levels (t_(70)_ = 1.71, p = 0.091, Fig 6A). Similarly, there were no differences in the quota size (t_(70)_ = 0.84, p = 0.404) and offtake levels (t_(70)_ = 1.25, p = 0.217) for African elephant between the two contrasting periods, 2004–2008 and 2009–2015 (Fig 6B). During the period 2004–2015, the recorded elephant offtake growth rate did not change (F_(11, 60)_ = 1.15, p = 0.340). Although there were no differences in the quota levels for greater kudu between the period 2004–2008 and 2009–2015 (t_(70)_ = 0.415, p = 0.679), the offtake levels recorded for the two periods differed significantly (t_(70)_ = 4.05, p < 0.000, Fig 6C). The quota size allocated for sable for the period 2004–2008 were lower than those for the 2009–2015 period (t_(70)_ = -4.77, p < 0.000). However, we did not record any difference in the offtake levels of sable between the same two periods (t_(70)_ = 0.52, p = 0.602, Fig 6D).

## Discussion

### Trophy Size and Age at Harvest Patterns

We hypothesized that trophy size for the four wild herbivores would decline over time due to continued selective harvesting pressure in Matetsi Safari Area. We recorded significant temporal declines in the trophy size of African elephant for the period 2004–2015 whilst that of Cape buffalo, greater kudu and sable did not change in Matetsi Safari Area. Our findings corroborate those by Crosmary, Loveridge [55] who reported similar trends in greater kudu and sable trophy size for the period 1979–2005 in Matetsi Safari Area. However, temporal declines in trophy size of wild herbivores over time have been reported in South Africa [56, 57] and Tanzania [44]. Declines in trophy size over time due to selective harvesting could be attributed to phenotypic plasticity [77] that may result due to a decline in abundance of big tuskers and individuals with big horns or tusks as these are mostly selected by hunters. However, our study examines data for a fairly short period (i.e., 11 years) and as such we may not attribute the observed changes to a possible genetic effect of selective pressures that favour expression of small horns or tusks [78–80].

Our results showed that African elephants’ age at harvest did not change over time though their trophy size declined. However, in this study for the period 2004–2015 we found sable age at harvest to have increased significantly contrary to the observations for the period 1979–2005 in the same area [55]. On the contrary, the trophy sizes for Cape buffalo and greater kudu have not changed for the period 2004–2015 in Matetsi Safari Area as reported in some countries, e.g., Tanzania [44] and South Africa [56, 57]. Most of the documented studies done on African elephant relates to illegal hunting effects on the tusk size instead of trophy hunting related issues and as such there were no comparative studies[81]. However, we note that most illegal hunting of elephants target the large tuskers and as such could have the same effect of trophy hunting.

Variations in trophy size and age at harvest could be as a result of several factors including (1) use of the fixed quota system that reduces the density or availability of old trophy individuals with the requisite trophy sizes, (2) lack of consistent age based trophy harvesting policy that penalises the harvesting of young individuals [46, 62, 64], (3) habitat quality heterogeneity that affect horn development and growth of trophy species [82, 83], and (4) possible effects of illegal harvesting that may vary with area and degree of protection [81]. However, there is uncertainty on the contribution of illegal harvesting activities on the trophy size of these herbivores as in some instances some poachers tend to select horn size for their kills in the same manner as regulated trophy hunting [24]. Most illegal hunters (i.e., subsistence hunters) who target plains game, e.g., greater kudu and sable tend to kill indiscriminately and do not select for trophy size [26, 27]. However, there has been worrying trends on illegal activities in Hwange region where illegal trophy hunting targeting African elephants has resorted to indiscriminate poisoning of large herds [23, 28].

The recorded Cape buffalo trophy size in Matetsi Safari Area might not necessarily indicate trophy quality, but rather a possible limitation of the SCI scoring system [84]. Though a decline in trophy size might suggest tendencies of unsustainable harvesting, a mature Cape buffalo bull has worn out horns that may produce low SCI tip to tip score [56, 84]. The SCI scoring system in Cape buffalo mostly results in high scores for green bulls or soft bossed bulls (immature bulls) which are still in their breeding prime thus undermining the best practices in Cape buffalo hunting where only mature bulls, past their breeding prime and has broomed horns [84, 85]. This maybe however different in the case of sable as there were declines in sable trophy size but with constant age at harvest in this study as also reported by Crosmary, Loveridge [55]. These variations may be due to habitat quality as a function of environmental heterogeneity that may influence resource allocation towards horn development and body growth over time [86–88]. Fluctuations in ecosystem productivity and habitat quality may result in cyclical trends in horn growth patterns observed in these species in Matetsi Safari Area as has been observed elsewhere [77].

### Temporal and Spatial Harvesting Patterns

The basis for an increase in sable quota size in Matetsi Safari Area for the period 2004–2015 is problematic given reports on a possible decline of sable in its usual range within the Hwange Conservation Area [50]. Our findings cast doubt on the sustainability of how the quota setting processes in this area as there are indications that the quota allocations are not based on real scientific data. There seems to be over-reliance on questionable and subjective personal opinions in the quota setting process which in actual sense is supposed to be based on scientific evidence and ecological principles [46, 47, 89]. The trends observed in this study seem to reflect on the persistent use of the ‘fixed quota’ approach that tends to encourage harvesting of young or prime breeding individuals as an attempt by concessionaires to utilize the entire fixed portion of the quota regardless of its sustainability [46]. We argue that viability of trophy hunting in this area over time may be compromised unless solemn trophy hunting policy changes are adopted and implemented. Failure to utilize the allocated quota may reflect on (1) loss of hunting destination competiveness due to a waning preference by trophy hunting clients to patronise a hunting destination or species to hunt, (2) possible decline in the abundance of suitable trophy individuals thus affecting the hunting success of hunted species over time. It is argued that the viability of trophy hunting in this area over time may be compromised unless a review on the current trophy hunting policy is done to ensure that a dynamic framework is adopted and implemented commensurate with the global trends in modern day conservation.

In this study, we report a spatial variation in trophy size attributes (i.e., age at harvest and trophy size) where Unit 6 had higher values for each of the attributes compared to Unit 1–5 except for Cape buffalo. Similar spatial variation in trophy size has been observed in the different provinces of South Africa [57]. However, as opposed to von Brandis and Reilly [56], our study was done in a more connected conservation area in the same region, where trophy hunting in Matetsi Area tends to utilize a shared, one source population given that there are no fences in this area. We argue that Unit 6 may be having different attributes compared to other Units because could be benefiting from a source and sink dynamics associated with the movement of the selected species from Zambezi National Park and Unit 7 where there is no hunting. Moreover, Unit 6 could be benefiting from individuals migrating from Chobe National Park within the KAZA TFCA network. On the contrary, in areas where hunting has been persistent, animals have been observed to evolve avoidance mechanisms to evade disturbances and hunting [42, 90]. Our observations in this study affirm the significance of sink and source dynamics of wildlife species between hunting and non-hunting areas within the KAZA TFCA network.

The historical and current trophy hunting activities in Matetsi might have shaped the anti-predator strategies of these herbivores thereby avoiding the hunting areas in favour of the neighbouring National Parks within the KAZA TFCA where there is no hunting as was observed in Tanzania [91]. African elephant and Cape buffalo have a tendency of migrating within large landscapes in search of water and feed resources and this may result in the variation of trophy sizes observed in these units [92–94]. Within the KAZA TFCA landscape, we argue that though these harvesting rates may be considered low in relation to the population estimates of these species: they may not be sustainable from a trophy size perspective if age restrictions and trophy size limits were to be imposed.

### Effect of Temporal Economic Status on Harvesting Patterns

In this study, there was no difference in the quota size allocation of Cape buffalo, African elephant, greater kudu and sable. Our results show that economic decline [95], may not affect the size of quota allocation levels in some hunting areas. However, it was evident that the economic decline also seriously incapacitated the Zimbabwe Parks and Wildlife Management Authority to do periodic surveys and monitoring programs which are critical in the quota setting process. Critical scientific data mostly obtained from aerial surveys in extensive and large protected areas were conducted in 2001 [96] and then recently in 2014 (*http://www.greatelephantcensus.com*). The quota sizes allocated during this period may have been based on previous experiences and individual opinions and not based on scientific principles as the MSY approach [39, 40].

Although Zimbabwe had an economic crisis, the Zimbabwe Parks and Wildlife Management Authority did not substantially increase the trophy quota size to increase revenue for the Parks and Wildlife Estate to cushion itself from the bad economy. Instead, the Zimbabwe Parks and Wildlife Management Authority adapted by re-assigning Unit 2 and 4 to fall under its hunting concessions in 2013 and 2012 respectively as was the case with Unit 5. Similar management interventions have been observed in Sengwa Wildlife Research Area, which also falls under the Zimbabwe Parks and Wildlife Management Authority, where hunting was also recently introduced. In an attempt to increase its revenue base, the Zimbabwe Parks and Wildlife Management Authority over and above its regulatory role as an Authority is also responsible for hunting in Unit 2, 4 and 5 of Matetsi Safari Area.

We hereby argue that to some extent, the Zimbabwe Parks and Wildlife Management Authority relied on trophy hunting as a possible source of income for its operations as has been argued elsewhere [5, 13, 62]. The Zimbabwe Parks and Wildlife Management Authority is confronted with the dual task of generating revenue and yet at the same time plays the regulatory role in trophy hunting and wildlife conservation issues in Zimbabwe. Self-regulating is always a problem as there is often questions on ‘who will police the regulator’ and may cause problems if the regulator looses focus and allows the economic benefits to take precedence over regulatory policy framework [97]. As Zimbabwe recovers from the economic doldrums, there is need to seriously consider re-looking at the model which is being used in Matetsi Safari Area Unit 2, 4, and 5. This would promote transparency and accountability in the sustainable use of wildlife resources through trophy hunting.

In this study, there was no difference in the offtake levels between 2004–2008 and 2009–2015 time periods confirming the suggestions by Leader-Williams and Hutton [15] that political instability or economic decline may not reduce the trophy hunters’ patronage to a hunting destination [95, 98]. However, with the restrictive policy on import bans of elephant trophy from Zimbabwe into USA [9] and other restrictions by some European countries [99, 100], it is clear that there will be a change in the proportion of hunters patronizing Zimbabwe for big game trophy hunting as the African elephant is one of the most sought for trophy species by most hunters. How this ban on the import of elephant trophies from Zimbabwe into USA as well as moratoriums by airlines in transporting such trophies would affect the trophy hunting in Zimbabwe and other southern African countries still need to be ascertained.

## Conclusions and Recommendations

We concluded that: (1) the effect of trophy hunting on size of horn or tusk size and age at harvest is species specific as it does not necessarily affect trophy size and the age at harvest of harvested herbivores, (2) quota size allocation may not reflect the trophy size and offtake levels over the time, and (3) political and economic performance may not necessarily affect the harvesting regime patterns though external influences though moratoriums may possibly reduce the offtake levels over time. Accordingly, trophy hunting may not necessarily lead to irreversible trophy size over time but requires systematic monitoring and sound management interventions for sustainability [101].

We recommend that conservationists and protected area managers may consider to: (1) emphasise the need for ecological principles in the quota setting process and in some cases reduce or temporarily stopping hunting (i.e., introducing fallow or resting hunting years on a rotational basis) of some species, (2) create temporal and spatial refuges to facilitate ‘trophy hunting rest’ for some species promote reproductive to the desirable phenotypic sizes such as trophy size[102], (3) introduce and firmly implement age based harvesting policies across all trophy hunted species instead of lions only, through enforcing penalties for harvesting below threshold age individuals [46, 89], and (4) introduce a variable trophy fee pricing system based on trophy size where the fees are determined on the trophy size [71, 101]. These measures could then be replicated at micro-scale and national level in other areas where trophy hunting is being practiced to ensure sustainability.

## Supporting Information

S1 File Trophy size and age at harvest of selected herbivores in Matetsi Safari Area, Zimbabwe. (XLSX)

S2 File Harvesting patterns of selected herbivores in Matetsi Safari Area, Zimbabwe. (XLSX)

## References

[1] Prins H, Grootenhuis JG, Dolan TT, editors. Wildlife conservation by sustainable use Netherlands: Springer Science & Business Media; 2012.

[2] Child B, editor. Parks in Transition:" Biodiversity, Rural Development and the Bottom Line": Routledge; 2013.

[3] Robinson JG. Ethical pluralism, pragmatism, and sustainability in conservation practice. Biological Conservation. 2011;144(3):958–65.

[4] Locke H, Dearden P. Rethinking protected area categories and the new paradigm. Environmental conservation. 2005;32(1):1–10.

[5] Lindsey PA, Alexander R, Frank LG, Mathieson A, Romanach SS. Potential of trophy hunting to create incentives for wildlife conservation in Africa where alternative wildlife-based land uses may not be viable. Animal Conservation. 2006;9(3):283–91. 10.1111/j.1469-1795.2006.00034.x

[6] Naidoo R, Weaver LC, Diggle RW, Matongo G, Stuart‐Hill G, Thouless C. Complementary benefits of tourism and hunting to communal conservancies in Namibia. Conservation Biology, In-press. 2016 10.1111/cobi.12643 26537845

[7] Lindsey PA, Frank LG, Alexander R, Mathieson A, Romanach SS. Trophy hunting and conservation in Africa: problems and one potential solution. Conservation Biology. 2007;21(3):880–3. Epub 2007/05/29. 10.1111/j.1523-1739.2006.00594.x . 17531065

[8] Lindsey P, Balme G, Funston P, Henschel P, Hunter LT. Life after Cecil: Channelling global outrage into funding for conservation in Africa. Conservation Letters. 2016.

[9] Di Minin E, Leader-Williams N, Bradshaw CJ. Banning Trophy Hunting Will Exacerbate Biodiversity Loss. Trends in Ecology & Evolution. 2016;31(2):99–102. 26746807 10.1016/j.tree.2015.12.006

[10] Costanza R. Ecosystem health and ecological engineering. Ecological Engineering. 2012;45:24–9.

[11] Padilla E. Intergenerational equity and sustainability. Ecological Economics. 2002;41(1):69–83.

[12] Sharma A. Ecotourism in J&K: vehicle to sustainable development. Review of Research Journal. 2014;3(8):ROR–741.

[13] Lindsey P, Roulet P, Romanach S. Economic and conservation significance of the trophy hunting industry in sub-Saharan Africa. Biological Conservation. 2007;134(4):455–69. 10.1016/j.biocon.2006.09.005

[14] Wilkie DS, Caprpenter JF. The potential role of safari hunting as a source of revenue for protected areas in the Congo Basin. Oryx. 1999;33:339–45.

[15] Leader-Williams N, Hutton JM, editors. Does extractive use provide opportunities to offset conflicts between people and wildlife? Cambridge: Cambridge University Press; 2005.

[16] Gandiwa E, Sprangers S, van Bommel S, Heitkönig IM, Leeuwis C, Prins HH. Spill-over effect in media framing: Representations of wildlife conservation in Zimbabwean and international media, 1989–2010. Journal for Nature Conservation. 2014;22(5):413–23.

[17] Craigie ID, Baillie JE, Balmford A, Carbone C, Collen B, Green RE, et al Large mammal population declines in Africa’s protected areas. Biological Conservation. 2010;143(9):2221–8.

[18] Ogutu J, Owen-Smith N, Piepho HP, Said M. Continuing wildlife population declines and range contraction in the Mara region of Kenya during 1977–2009. Journal of Zoology. 2011;285(2):99–109.

[19] Whitman K, Starfield M, Quadling S, Packer C. Sustainable trophy hunting of African lions. Nature. 2004;428:175–8. 10.1038/nature02395 14990967

[20] Wroe S, Field J, Fullagar R, Jermin LS. Megafaunal extinction in the late Quaternary and the global overkill hypothesis. Alcheringa. 2004;28(1):291–331.

[21] Martin PS. African and Pleistocene overkill. Nature 1966;212:339–42. 10.1038/2121615a0 21105543

[22] Gandiwa E. Top-down and bottom-up control of large herbivore populations: a review of natural and human-induced influences. Tropical Conservation Science. 2013;6(4):493–505.

[23] Muboko N, Gandiwa E, Muposhi VK, Tarakini T. Illegal hunting and protected areas: Tourist perceptions on wild animal poisoning in Hwange National Park, Zimbabwe. Tourism Management. 2016;52:170–2.

[24] Martin A, Caro T. Illegal hunting in the Katavi-Rukwa ecosystem. African Journal of Ecology. 2013;51(1):172–5.

[25] Marealle WN, Fossøy F, Holmern T, Stokke BG, Røskaft E. Does illegal hunting skew Serengeti wildlife sex ratios? Wildlife Biology. 2010;16(4):419–29.

[26] Gandiwa E, Zisadza-Gandiwa P, Mango L, Jakarasi J. Law enforcement staff perceptions of illegal hunting and wildlife conservation in the Gonarezhou National Park, southeast Zimbabwe. Tropical Ecology. 2014;55:119–27.

[27] Gandiwa E. Preliminary assessment of illegal hunting by communities adjacent to the northern Gonarezhou National Park, Zimbabwe. Tropical Conservation Science. 2011;4(4):445–67.

[28] Muboko N, Muposhi V, Tarakini T, Gandiwa E, Vengesayi S, Makuwe E. Cyanide poisoning and African elephant mortality in Hwange National Park, Zimbabwe: a preliminary assessment. Pachyderm. 2014;(55):92–4.

[29] Whitman K, Starfield AM, Quadling HS, Packer C. Sustainable trophy hunting of African lions. Nature. 2004;428(6979):175–8. 10.1038/nature02395 14990967

[30] Wilson EO. Threats to biodiversity. Scientific American. 1989;261(3):108–16.

[31] Riggio J, Jacobson A, Dollar L, Bauer H, Becker M, Dickman A, et al The size of savannah Africa: a lion’s (*Panthera leo*) view. Biodiversity and Conservation. 2013;22(1):17–35.

[32] Seabrook L, McAlpine C, Baxter G, Rhodes J, Bradley A, Lunney D. Drought-driven change in wildlife distribution and numbers: a case study of koalas in south west Queensland. Wildlife Research. 2011;38(6):509–24.

[33] Dudley J, Criag G, Gibson D, Haynes G, Klimowicz J. Drought mortality of bush elephants in Hwange National Park, Zimbabwe. African Journal of Ecology. 2001;39(2):187–94.

[34] Chamaillé-Jammes S, Fritz H, Murindagomo F. Detecting climate changes of concern in highly variable environments: Quantile regressions reveal that droughts worsen in Hwange National Park, Zimbabwe. Journal of Arid Environments. 2007;71(3):321–6.

[35] Fryxell JM, Packer C, McCann K, Solberg EJ, Sæther B-E. Resource management cycles and the sustainability of harvested wildlife populations. Science. 2010;328(5980):903–6. 10.1126/science.1185802 20466934

[36] Morrill WI. The tourist safari hunter’s role in conservation. Paper prepared for Safari Club International, Herndon, VA. 1993.

[37] Cooley HS, Wielgus RB, Koehler GM, Robinson HS, Maletzke BT. Does hunting regulate cougar populations? A test of the compensatory mortality hypothesis. Ecology. 2009;90(10):2913–21. 19886499 10.1890/08-1805.1

[38] Songorwa AN. Community-based wildlife management (CWM) in Tanzania: Are the communities interested? World development. 1999;27(12):2061–79.

[39] Milner-Gulland EJ, Bunnefeld N, Proaktor G. The science of sustainable hunting In: Dickson B, Hutton J, Adams B, editors. Recreational hunting, conservation and rural livelihoods. Oxford: John Wiley & Sons; 2009 p. 75–93.

[40] Sutherland WJ. Sustainable exploitation: a review of principles and methods. Wildlife Biology. 2001;7(3):131–40.

[41] Crosmary WG, Côté S, Fritz H. The assessment of the role of trophy hunting in wildlife conservation. Animal Conservation. 2015;18(2):136–7.

[42] Muposhi VK, Gandiwa E, Makuza SM, Bartels P. Trophy hunting and perceived risk in closed ecosystems: flight behaviour of three gregarious African ungulates in a semi-arid tropical savanna. Austral Ecology. In press.

[43] Damm GR. Recreational trophy hunting: "What do we know and what should we do?". In: Baldus RD, Damm GR, Wollscheid K, editors. Best practices in sustainable hunting-A guide to bes practices from around the world. Hungary: International Council for Game and Wildlife Conservation; 2008 p. 5–11.

[44] Wilfred P. Trophy hunting and trophy size in Ugulla Game Reserve, Western Tanzania. Tanzanian Journal Science. 2012;38(2):111–22.

[45] Selier SAJ, Page BR, Vanak AT, Slotow R. Sustainability of elephant hunting across international borders in southern Africa: A case study of the greater Mapungubwe Transfrontier Conservation Area. The Journal of Wildlife Management. 2014;78(1):122–32.

[46] Lindsey PA, Balme GA, Funston P, Henschel P, Hunter L, Madzikanda H, et al The trophy hunting of African lions: Scale, current management practices and factors undermining sustainability. Plos One. 2013;8(9):e73808 10.1371/journal.pone.0073808 24058491 PMC3776777

[47] Loveridge AJ, Reynolds JC, Milner-Gulland E. Does sport hunting benefit conservation In: Macdonald D, Service K, editors. Key topics in conservation biology. UK: Blackwell Publishing Ltd; 2007 p. 222.

[48] Burkhardt-Holm P, Giger W, GUttinger H, Ochsenbein U, Peter A, Scheurer K, et al Where have all the fish gone? Environmental Science & Technology. 2005;39(21):441A–7A. 10.1021/es053375z 16294843

[49] Sarvala J, Langenberg V, Salonen K, Chitamwebwa D, Coulter G, Huttula T, et al Fish catches from Lake Tanganyika mainly reflect changes in fishery practices, not climate. Verhandlungen Internationale Vereinigung für theoretische und angewandte Limnologie. 2006;29:1182–8.

[50] Crosmary W-G, Chamaillé-Jammes S, Mtare G, Fritz H, Côté SD. Decline of sable antelope in one of its key conservation areas: the greater Hwange ecosystem, Zimbabwe. African Journal of Ecology. 2015;53(2):194–205. 10.1111/aje.12207

[51] Crosmary WG, Côté SD, Fritz H. Does trophy hunting matter to long-term population trends in African herbivores of different dietary guilds? Animal Conservation. 2015;18(2):117–30. 10.1111/acv.12144

[52] Nelson MP, Bruskotter JT, Vucetich JA, Chapron G. Emotions and the ethics of consequence in conservation decisions: Lessons from Cecil the Lion. Conservation Letters. 2016 10.1111/conl.12232

[53] Pérez JM, Serrano E, González-Candela M, León-Vizcaino L, Barberá GG, Simón MAd, et al Reduced horn size in two wild trophy-hunted species of Caprinae. Wildlife Biology. 2011;17(1):102–12. 10.2981/09-102

[54] Chomba C, Senzota R, Chabwela H, Nyirenda V. Lion Hunting and Trophy Quality Records in Zambia for the Period 1967–2000: Will the Trends in Trophy Size Drop as Lion Population Declines? Open Journal of Ecology. 2014;4(04):182–95.

[55] Crosmary WG, Loveridge AJ, Ndaimani H, Lebel S, Booth V, Côté SD, et al Trophy hunting in Africa: long-term trends in antelope horn size. Animal Conservation. 2013;16(6):648–60. 10.1111/acv.12043

[56] von Brandis RG, Reilly BK. A temporal analysis of trophy quality in South Africa: has trophy quality changed over time. South African Journal of Wildlife Research. 2007;37(2):153–8.

[57] von Brandis RG, Reilly BK. Spatial variation in trophy quality of popular hunted ungulate species in South Africa. South African Journal of Wildlife Research. 2008;38(1):17–21. 10.3957/0379-4369-38.1.17

[58] Hedrick PW. Rapid decrease in horn size of bighorn sheep: environmental decline, inbreeding depression, or evolutionary response to trophy hunting? Journal of Heredity. 2011:esr082. 10.1093/jhered/esr082 21900210

[59] Barnett R, Patterson C. Sport Hunting in the Southern African Development Community (SADC) Region: An overview Johannesburg, South Africa: TRAFFIC East/Southern Africa; 2005.

[60] Buckley R, Mossaz A. Hunting tourism and animal conservation. Animal Conservation. 2015;18(2):133–5.

[61] Lindsey P, Alexander R, Balme G, Midlane N, Craig J. Possible Relationships between the South African Captive-Bred Lion Hunting Industry and the Hunting and Conservation of Lions Elsewhere in Africa. South African Journal of Wildlife Research. 2012;42(1):11–22. 10.3957/056.042.0103

[62] Lindsey PA, Balme GA, Booth VR, Midlane N. The significance of African lions for the financial viability of trophy hunting and the maintenance of wild land. PloS one. 2012;7(1):e29332 10.1371/journal.pone.0029332 22247772 PMC3256150

[63] Balme GA, Hunter LT, Goodman P, Ferguson H, Craigie J, Slotow R. An adaptive management approach to trophy hunting of leopards Panthera pardus: a case study from KwaZulu-Natal, South Africa Biology and conservation of wild felids. Oxford, United Kingdom: Oxford University Press; 2010 p. 341–52.

[64] Balme GA, Hunter L, Braczkowski AR. Applicability of age-based hunting regulations for African leopards. PloS One. 2012;7(4):e35209 10.1371/journal.pone.0035209 22493739 PMC3320874

[65] de Garine-Wichatitsky M, Miguel E, Mukamuri B, Garine-Wichatitsky E, Wencelius J, Pfukenyi DM, et al Coexisting with wildlife in transfrontier conservation areas in Zimbabwe: Cattle owners’ awareness of disease risks and perceptions of the role played by wildlife. Comparative Immunology, Microbiology and Infectious Diseases. 2013;36(3):321–32. 10.1016/j.cimid.2012.10.007 23219685

[66] Cumming D. Constraints to conservation and development success at the wildlife–livestock–human interface in Southern African transfrontier conservation areas: a preliminary review. Report to the Wildlife Conservation Society (WCS). 2011. Available: http://www.wcs.ahead.org/kaza/constraints_to_cons_&_dev_success_082311.pdf. Accessed 3 March 2013.

[67] Ndaimani H, Murwira A, Kativu S. Comparing terrain and vegetation- based visibility for explaining sable flight behaviour in a Southern African savanna. Geocarto International. 2013;28(2):130–43. 10.1080/10106049.2012.677481

[68] Chenje M, Sola L, Pateczny D, editors. The state of Zimbabwe’s environment Harare, Zimbabwe: Ministry of Mines, Environment and Tourism; 1998.

[69] Chamaille‐Jammes S, Fritz H, Murindagomo F. Spatial patterns of the NDVI–rainfall relationship at the seasonal and interannual time scales in an African savanna. International Journal of Remote Sensing. 2006;27(23):5185–200.

[70] Muposhi VK, Gandiwa E, Chemura A, Bartels P, Makuza SM, Madiri TH. Habitat heterogeneity variably influences habitat selection by wild berbivores in a semi-arid tropical savanna ecosystem. PLoS ONE, 11(9): e0163084 2016 10.1371/journal.pone.0163084 27680673 PMC5040439

[71] Johnson PJ, Kansky R, Loveridge AJ, Macdonald DW. Size, rarity and charisma: valuing African wildlife trophies. PloS one. 2010;5(9):e12866 10.1371/journal.pone.0012866 20877564 PMC2943918

[72] Laws R. Age criteria for the African elephant. African Journal of Ecology. 1966;4(1):1–37.

[73] Grimsdell J. Age determination of the African buffalo, Syncerus caffer Sparrman. African Journal of Ecology. 1973;11(1):31–53.

[74] Spinage C. A review of the age determination of mammals by means of teeth, with especial reference to Africa. African Journal of Ecology. 1973;11(2):165–87.

[75] Rist J, Milner-Gulland EJ, Cowlishaw G, Rowcliffe M. Hunter Reporting of Catch Per Unit Effort as a Monitoring Tool in a Bushmeat-Harvesting System. Conservation Biology. 2010;24(2):489–99. 10.1111/j.1523-1739.2010.01470.x 20491849

[76] Milner JM, Bonenfant C, Mysterud A, Gaillard JM, Csanyi S, Stenseth NC. Temporal and spatial development of red deer harvesting in Europe: biological and cultural factors. Journal of Applied Ecology. 2006;43(4):721–34.

[77] Loehr J, Carey J, O’HARA R, Hik D. The role of phenotypic plasticity in responses of hunted thinhorn sheep ram horn growth to changing climate conditions. Journal of Evolutionary Biology. 2010;23(4):783–90. 10.1111/j.1420-9101.2010.01948.x 20163506

[78] Fenberg PB, Kaustuv R. Ecological and evolutionary consequences of size-selective harvesting: how much do we know? Molecular ecology. 2008;17:209–20. 10.1111/j.1365-294X.2007.03522.x 17868288

[79] Mysterud A. Selective harvesting of large mammals: how often does it result in directional selection? Journal of Applied Ecology. 2011;48(4):827–34. 10.1111/j.1365-2664.2011.02006.x

[80] Coltman DW, O’Donoghue P, Jorgenson JT, Hogg JT, Strobeck C, Festa-Bianchet M. Undesirable evolutionary consequences of trophy hunting. Nature. 2003;426:655–7. 14668862 10.1038/nature02177

[81] Chiyo PI, Obanda V, Korir DK. Illegal tusk harvest and the decline of tusk size in the African elephant. Ecology and Evolution. 2015;5(22):5216–29. 30151125 10.1002/ece3.1769 PMC6102531

[82] Vanpé C, Morellet N, Kjellander P, Goulard M, Liberg O, Hewison A. Access to mates in a territorial ungulate is determined by the size of a male's territory, but not by its habitat quality. Journal of Animal Ecology. 2009;78(1):42–51. 10.1111/j.1365-2656.2008.01467.x 18752539

[83] Landete-Castillejos T, Estevez J, Martínez A, Ceacero F, Garcia A, Gallego L. Does chemical composition of antler bone reflect the physiological effort made to grow it? Bone. 2007;40(4):1095–102. 10.1016/j.bone.2006.11.022 17239669

[84] Gandy SE, Reilly BK. Alternative trophy measuring techniques for African buffalo. Koedoe. 2004;47(1):119–24.

[85] Taylor W. The Influence of trophy measurement in Cape Buffalo. African Indaba. 2007;5(3).

[86] Ramanzin M, Sturaro E. Habitat quality influences relative antler size and hunters’ selectivity in roe deer. European Journal of Wildlife Ressearch. 2013;60(1):1–10. 10.1007/s10344-013-0744-5

[87] Festa-Bianchet M, Coltman DW, Turelli L., Jorgenson TT. Relative allocation to horn and body growth in bighorn rams varies with resource availability. Behavioral Ecology. 2004;15:305–12.

[88] Schmidt JI, Ver Hoef JM, Terry Bowyer R. Antler size of Alaskan moose Alces alces gigas: effects of population density, hunter harvest and use of guides. Wildlife Biology. 2007;13(1):53–65.

[89] Loveridge AJ, Searle AW, Murindagomo F., Macdonald DW. The impact of sport-hunting on the population dynamics of an African lion population in a protected area. Biological Conservation 2007;134:548–58. 10.1016/j.biocon.2006.09.010

[90] Malo JE, Acebes P, Traba J. Measuring ungulate tolerance to human with flight distance: a reliable visitor management tool? Biodiversity and Conservation. 2011;20(14):3477–88.

[91] Nyahongo JW. Flight initiation distances of five herbivores to approaches by vehicles in the Serengeti National Park, Tanzania. African Journal of Ecology. 2008;46(2):227–9.

[92] Douglas-Hamilton I, Krink T, Vollrath F. Movements and corridors of African elephants in relation to protected areas. Naturwissenschaften. 2005;92(4):158–63. 10.1007/s00114-004-0606-9 15770465

[93] Georgiadis N, Bischof L, Templeton A, Patton J, Karesh W, Western D. Structure and history of African elephant populations: I. Eastern and southern Africa. Journal of Heredity. 1994;85(2):100–4. 7910176 10.1093/oxfordjournals.jhered.a111405

[94] Naidoo R, Du Preez P, Stuart-Hill G, Jago M, Wegmann M. Home on the range: factors explaining partial migration of African buffalo in a tropical environment. PLoS One. 2012;7(5):e36527 10.1371/journal.pone.0036527 22570722 PMC3343005

[95] Gandiwa E, Heitkönig IMA, Lokhorst AM, Prins HHT, Leeuwis C. Illegal hunting and law enforcement during a period of economic decline in Zimbabwe: A case study of northern Gonarezhou National Park and adjacent areas. Journal for Nature Conservation. 2013;21: 133–42.

[96] Dunham KM. Aerial census of elephants and other large herbivores in the north-west Matebeleland, Zimbabwe: 2001 Occassional Paper 6 ed. Harare: WWF-SARPO; 2002 p. 77.

[97] Short JL, Toffel MW. Coerced confessions: Self-policing in the shadow of the regulator. Journal of Law, Economics, and Organization. 2008;24(1):45–71.

[98] Lindsey PA, Romanach S, Tambling CJ, Chartier K, Groom R. Ecological and financial impacts of illegal bushmeat trade in Zimbabwe. Oryx. 2011;45(01):96–111.

[99] Knapp A. A review of the European Union’s import policies for hunting trophies. A TRAFFIC Europe Report for the European Commission. Brussels, Belgium. 2007. p. 81.

[100] UNEP-WCMC. Assessing potential impacts of trade in trophies imported for hunting purposes to the EU-27 on conservation status of Annex B species. Part 2: Discussion and case studies UNEP-WCMC Cambridge: UK; 2013 p. 34.

[101] Rivrud IM, Sonkoly K, Lehoczki R, Csányi S, Storvik GO, Mysterud A, et al Hunter selection and long-term trend (1881–2008) of red deer trophy sizes in Hungary. Journal of Applied Ecology. 2013;50(1):168–80. 10.1111/1365-2664.12004.

[102] Hengeveld PE, Festa‐Bianchet M. Harvest regulations and artificial selection on horn size in male bighorn sheep. The Journal of Wildlife Management. 2011;75(1):189–97.

